# The Role of MicroRNAs in Diabetes-Related Oxidative Stress

**DOI:** 10.3390/ijms20215423

**Published:** 2019-10-31

**Authors:** Mirza Muhammad Fahd Qadir, Dagmar Klein, Silvia Álvarez-Cubela, Juan Domínguez-Bendala, Ricardo Luis Pastori

**Affiliations:** 1 Diabetes Research Institute, University of Miami Miller School of Medicine, Miami, FL 33136, USA; fahd.qadir@med.miami.edu (M.M.F.Q.); dklein@med.miami.edu (D.K.); salvarez@med.miami.edu (S.Á.-C.); 2 Department of Cell Biology and Anatomy, University of Miami Miller School of Medicine, Miami, FL 33136, USA; 3 Department of Surgery, University of Miami Miller School of Medicine, Miami, FL 33136, USA; 4 Department of Medicine, Division of Metabolism, Endocrinology and Diabetes, University of Miami Miller School of Medicine, Miami, FL 33136, USA

**Keywords:** diabetes, beta cells, oxidative stress, microRNAs

## Abstract

Cellular stress, combined with dysfunctional, inadequate mitochondrial phosphorylation, produces an excessive amount of reactive oxygen species (ROS) and an increased level of ROS in cells, which leads to oxidation and subsequent cellular damage. Because of its cell damaging action, an association between anomalous ROS production and disease such as Type 1 (T1D) and Type 2 (T2D) diabetes, as well as their complications, has been well established. However, there is a lack of understanding about genome-driven responses to ROS-mediated cellular stress. Over the last decade, multiple studies have suggested a link between oxidative stress and microRNAs (miRNAs). The miRNAs are small non-coding RNAs that mostly suppress expression of the target gene by interaction with its 3’untranslated region (3′UTR). In this paper, we review the recent progress in the field, focusing on the association between miRNAs and oxidative stress during the progression of diabetes.

## 1. Introduction

Diabetes, which affects approximately 422 million people worldwide, is a disease characterized by the loss of glycemic control, which causes side effects such as polyuria, glycosuria, weight loss, neuropathies, retinopathy, and renal plus vascular diseases. Because diabetes results in the loss of glucose homeostasis, it is associated with high morbidity and mortality [1]. The most prevalent forms of this disease are Type 1 (T1D) and Type 2 diabetes (T2D). Both types are characterized by hyperglycemia due to either insufficient insulin production (T1D) or loss of cellular sensitivity to insulin, known as insulin resistance (T2D). Insulin-producing beta cells reside in the pancreas within clusters of endocrine cells called “Islets of Langerhans”. Islets are dispersed throughout the pancreas, representing around 2% of the overall pancreatic tissue [2]. Beta cells are essential for blood glucose homeostasis. Their dysregulation is linked to both forms of diabetes. In T1D, the primary targets of autoimmunity are beta cells [3]. In T2D, insulin resistance (i.e., the inability of cells to respond to insulin to take up glucose) leads to excessive insulin production by beta cells, resulting in their exhaustion and eventual death [4]. Strong evidence indicates that T2D is associated with a deficit in beta cell mass [5], which leads to long lasting inefficient glycemic control leading to toxic amount of glucose. Hyperglycemia is responsible for the development of severe complications such as microvascular, neuropathic, and macrovascular problems, which affect the quality and expectancy of life [6,7]. 

Since beta cells have notoriously low proliferating rates in adults, replenishing beta cell mass remains one of the greatest challenges of modern biology [8,9]. Even a partial restoration of insulin production in the pancreas could be therapeutically sufficient, judging by the fact that even after 80% loss of beta cell mass, T1D patients remain asymptomatic [10]. Although each of the two diabetes types has a different etiology, they are both greatly affected by cellular oxidative stress. On the one hand, oxidative stress in T1D originates from T cell-mediated autoimmunity targeting beta cells through the generation of proinflammatory cytokines. In addition, low tissue expression of antioxidative enzymes and antioxidative agents make affected individuals vulnerable to damage induced by reactive oxygen species (ROS) and reactive nitrogen species (RNS) originating from hypoxia or cytokine-mediated oxidative stress. A well-balanced equilibrium between oxidative molecules and antioxidative defenses is critical for physiological cell functions. On the other hand, type 2 diabetes is a metabolic syndrome where a group of conditions such as hypertension, glucose intolerance, insulin resistance, obesity, and dyslipidemia result in cellular oxidative stress across tissues [11,12]. Specifically, abdominal obesity has been shown to be a source of proinflammatory cytokines and, consequently, leads to insulin resistance.

Numerous studies have recently reported a strong link between oxidative stress and microRNAs (miRNAs). MiRNAs are post-transcriptional regulators, approximately 18 to 23 nucleotides long, that suppress gene expression by specific interaction with target genes [13]. The miRNAs have a role in controlling cellular redox homeostasis between highly reactive oxidative and antioxidative species. Current reports show that changes in miRNA levels contribute to persistent cellular oxidative stress, eventually leading to the development of diseases. Publications over the last few years increasingly support the link between miRNAs and oxidative stress in diabetes. A better understanding of the molecular mechanisms influencing the relationship between miRNAs and oxidative stress in diabetes could be useful to the development of therapeutic approaches that improve beta cell survival under metabolic stress. In this paper, we review the progress made in this field, describing mechanistic miRNA-driven gene regulation during oxidative stress and diabetes progression.

## 2. Overview of MicroRNA Biology: MiRNA Regulation and Their Role in Islets and Diabetes 

The discovery of microRNA (miRNA) over twenty-five years ago revolutionized the field of cell biology and molecular biology. The first well-characterized small RNAs were lin-4 and let-7 [14,15,16], both of which have been found to be involved in control of early development, while let-7 has been found highly conserved across animal species [17]. According to a conservative analysis from ENCODE (Encyclopedia of DNA Elements) [18], an international consortium funded by the National Human Genome Research Institute (NHGRI) to study the human genome, 62% of the genome bases are transcribed into RNA of more than 200 bases long, of which only 5% corresponds to exons. Therefore, most of the transcribed RNA does not code for proteins and is designated as non-protein coding RNA (ncRNA). MiRNAs, a subset of ncRNAs, are small single stranded gene products of 18 to 23 nts, with an important role in post-transcriptional regulation of gene expression [13,19]. Almost half of the human miRNA genes are located in intergenic regions of the genome. Most of the other half are located in intronic regions of protein-coding genes, whereas some are found within exons [20]. The most common miRNA biogenesis pathway is known as the canonical pathway, although some miRNAs take alternative biogenesis routes [21,22]. In the canonical pathway, miRNA genes are transcribed by RNA polymerase II (Pol-II) to primary miRNAs (pri-miRNAs), which are processed in the nucleus by a microprocessor complex composed of human ribonuclease III (Drosha) and the DGCR8 (DiGeorge syndrome critical region 8) to a pre-miR stem loop precursor of approximately 60 to 70 nt [13,23]. The pre-miRNA stem loop is actively transported to cytoplasm by exportin 5, where it is cleaved by Dicer, another member of the ribonuclease III protein family, into approximately 18 to 23 nucleotide double-stranded mature miRNA [13]. One strand arises from the 5′ end of the stem-loop and the other strand from the 3′ end, termed -5p and -3p, respectively. The miRNA is then incorporated into a ribonucleoprotein complex known as RISC (RNA-induced silencing complex) containing the essential silencing protein Argonaute 2 (Argo2) [24]. Argonautes belong to a highly conserved protein family. Together with small RNAs, such as miRNAs, they form ribonucleoprotein complexes (RNPs) that regulate post-transcriptional gene pathways. If the complementarity with the target mRNA is extensive, as is the case for the homeobox HOXB8 mRNA and miR-196, the Argonaute protein cleaves the mRNA [25]. However, in eukaryotes, the most frequent forms of silencing are by inhibition of translation or mRNA destabilization by polyA shortening [26].

**Figure 1:**
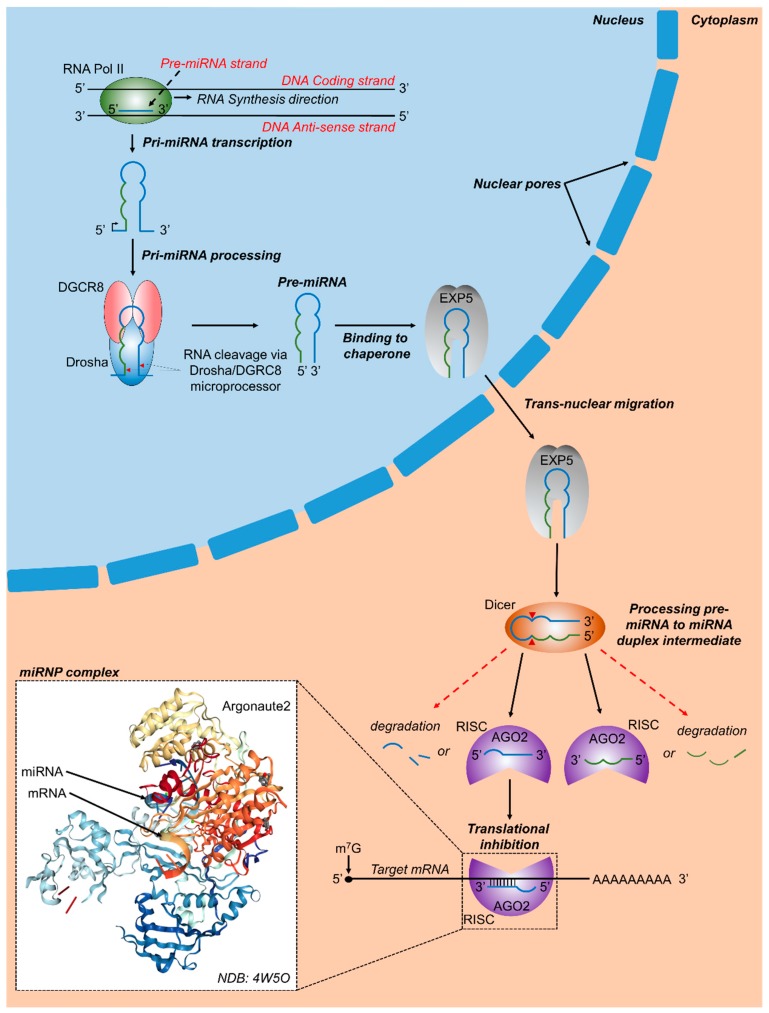
Canonical microRNA biogenesis and RNA targeting. In vertebrates, RNA polymerase-II transcribes primary miRNA genes (pri-miRNAs), which contain a hairpin-loop along with 5′ and 3′ flanking regions. DGCR8 (DiGeorge critical region 8) and a Drosha molecule combine to form the microprocessor complex which binds with pri-miRNA and cleaves it at specific sites (red arrowheads). The resulting precursor miRNA (pre-miRNA) contains a phosphate on its 5′ end and a hydroxyl group on its 3′ end along with a 2 to 3 nucleotide over-hang. Subsequently, the nuclear chaperone Exportin 5 (EXP5) binds to pre-miRNA molecules and transports pre-miRNA molecules to the cytoplasm via transnuclear migration, where Dicer, another RNAse III enzyme, binds to pre-miRNA molecules, cleaves them at specific regions, and releases a miRNA duplex intermediate. Argonaute 2 (AGO2) and other proteins assemble with miRNA molecules released from the miRNA duplex intermediate, together forming the RNA induced silencing complex (RISC). The 3′ or 5′ miRNA containing RISCs may bind to target regions and either result in translational repression, mRNA degradation, or in some cases translational activation. Inset shows a crystal structure of human Argonaute 2 bound to a guide and target RNA [30].

Only the active mature RNA strand, known as a guide strand, is preserved and loaded on RISC, while the other complementary strand, designated as * strand, and known as a passenger strand, is degraded [24]. Many miRNAs retain both 5′ and 3′ strands, which are then incorporated into RISC complexes, generating miR-5p, as well as miR-3p. The choice of miR-5p or -3p as active mature miRNAs depends mostly on cell type [27]. It appears that the decision to select the guide strand from the miRNA duplex generated by Dicer is partly due to thermodynamics considerations. The strand with the weakest binding at its 5′ end is more likely to become the guide strand. In many human miRNAs, the guide strand is U-biased at the 5′ end with an excess of purines, while the passenger strand is C-biased with an excess of pyrimidines. Proteins such as Dicer, Argo2, and others participate in this decision as well. However, the mechanism is basically unknown [28]. The miRNA leads the RISC to a target mRNA. The single strand miRNA-RISC-Argo2 complex principally functions to inhibit target gene expression through recognition of partially complementary sequences in messenger RNA (mRNA), thus regulating mRNA translation by inhibiting gene expression and protein translation. The recognition sequence on the target mRNA is usually found at the 3′ UTR and is recognized by the “seed” sequence, two to eight nucleotides long, located at the 5′ domain of the miRNA. The MiRNAs target specific genes, which in turn may be targeted by many different miRNAs, hence regulating entire critical cellular expression networks (Figure 1). 

It has been estimated that over 60% of human protein-coding genes are targets of miRNAs [29].

To date, the human genome contains 1917 annotated hairpin precursors, and 2654 mature sequences which are annotated in the Wellcome Trust Sanger Institute miRNA database [31] (http://www.mirbase.org/cgi-bin/mirna_summary.pl?org=hsa). miRNAs play a fundamental role in regulation of gene expression in key biological events such as cell proliferation, differentiation, death, and malignant transformation [13,32,33,34,35]. Consequently, impairment of miRNA expression is the underlying cause of many diseases. The miRNAs are mostly intracellular, but they are also found circulating in the body fluids, such as plasma or urine. They are extremely stable in human fluids, and therefore are well suited as clinical biomarkers [36]. They are protected from nucleases either by forming ribonucleoprotein particles (RNPs) with RNA-interacting proteins such as the RISC protein Ago2 or enclosed in extracellular vehicles (EVs) such as exosomes, present in and released by the majority of cell types [37]. The exosome-mediated transfer of mRNAs and miRNAs is a mechanism of cellular communication and genetic exchange among cells. The biogenesis, mode of action and suitability of circulating miRNAs as biomarkers for several diseases, is a hot research topic in biomedicine. Numerous studies suggest that miRNAs have an active role in pancreas organogenesis and in islet function [38,39,40,41,42]. An important study regarding miRNAs and their role in islet development is a report on the deletion of *Dicer1* in pancreatic progenitors. *Dicer1* is an enzyme involved in miRNA maturation, and its loss results in a marked reduction of endocrine cells [40]. Likewise, deletion of *Dicer1* in embryonic beta cells results in fewer beta cells, and impaired glucose tolerance [43,44]. There is evidence that miRNAs are involved in the pathogenesis of diabetes. Comprehensive reviews describing miRNAs in the context of T1D, T2D, and other diabetes models have recently become available. Furthermore, the role of miRNAs in tissues targeted by insulin, and in healthy or stressed islets, have been reported [45,46,47,48]. We have previously identified a subset of miRNAs differentially expressed in developing human islets, in human developing pancreas, and in alpha and beta cells of adult human islets [49,50,51,52]. These observations set the stage for studies to specifically assess the role of miRNAs and their target molecules in endocrine differentiation. In fact, many studies, including ours, identified individual miRNAs enriching endocrine tissue such as, miR-375 and miR-7, with the role in beta cell differentiation and function [53,54,55,56,57]. The same miRNAs have an important role in in vitro human stem cell differentiation into beta cells [58,59,60,61]. On the basis of the information presented above, it can be implied that oxidative stress affecting deregulation of miRNA networks, which is important for acquisition and maintenance of beta cell identity or proper cellular function and metabolism, contributes to the development of diabetes [62]. 

## 3. Overview of Oxidative Stress in Glucose Metabolism

The term oxidative stress refers to an imbalance between cellular oxidants and antioxidants [63,64]. Oxidative stress can be classified into the following two major groups: Endogenous (mitochondrial, peroxisomes, lipoxygenases, NADPH oxidase (NOX), and cytochrome P450) and exogenous (UV and ionizing radiation, chemotherapeutics, inflammatory cytokines, and environmental toxins). Oxidative stress is an accumulation of reactive oxygen species (ROS) above physiological levels, where ROS molecules oxidize cellular components stochastically, leading to progressive cellular damage. Under physiological conditions, the utmost ROS generation occurs in mitochondria, accounting for the transformation of 1% to 2% of oxygen molecules into superoxide anions [65]. Adenosine 5′-triphosphate (ATP) molecules are the major cellular energy currency. Generation of ATP in mitochondria, results in the production of ROS which occurs on two occasions with electron transport chain, at complex-I (NADH dehydrogenase) and at complex-III (ubiquinone-cytochrome c reductase). ATPs are first generated in the breakdown of glucose molecules during glycolysis. Glycolysis of one glucose molecule yields two pyruvate molecules with a net gain of only two ATP molecules. The greatest contributor to ATP production is the subsequent metabolism of pyruvate in the mitochondria through the tricarboxylic acid cycle, followed by oxidation of its energy mediators, NADH and FADH2, in the electron transport chain. In this process, known as oxidative phosphorylation, electrons are transferred from electron donors to electron acceptors via redox reactions. Oxidative phosphorylation, hypothetically, generates a maximum of 36 ATP molecules per glucose molecule. Oxygen is the final electron acceptor, generating H_2_O. Incomplete transfer of electrons to oxygen results in the production of reactive oxygen species (ROS) such as superoxide or peroxide anions. Superoxide is rapidly converted [66] into peroxide (H_2_O_2_) by the enzyme superoxide dismutase (SOD). Hydrogen peroxide, in turn, is either neutralized to H_2_O and O_2_ by glutathione peroxidase (Gpx, in the mitochondria), or detoxified by catalase in peroxisomes. Increased levels of Cu (copper) and Fe (iron) and significantly decreased levels of Zn (zinc) in the serum of T2D patients and their first degree relatives (FDR) could be either triggering factors for the development of diabetes or a consequence of the illness [67]. H_2_O_2_ can be converted into highly reactive radical hydroxyl (HO·), the neutral form of the hydroxide ion, via the Fenton reaction. Hydroxyl radicals target the DNA base deoxyguanosine with great efficiency [65,68]. 

A discrete amount of ROS is necessary for efficient cellular physiological function. For example, ROS are one of the metabolic signals for insulin secretion [69] and play an essential role as promoter of natural defenses [70,71]. If the production of ROS during mitochondrial oxidative phosphorylation is not well balanced by antioxidative activity, ROS become toxic [66]. Even though oxidative phosphorylation is a significant contributor to the formation of ROS, recent studies have identified other cellular sources of ROS, such as peroxisomes, endoplasmic reticulum, and plasma membrane, which could contribute to tissue oxidative damage [72]. ROS are free radicals and, because they have unpaired valence electrons, they are extremely reactive with many electron donor molecules such as membrane lipids, proteins, and DNA, leading to potential toxicity. Overproduction of ROS causes oxidative stress associated with numerous diseases and aging.

The interaction of ROS with the cell membrane’s polyunsaturated fatty acids generates a lipid peroxidation chain reaction with the production of toxic and highly reactive aldehyde metabolites such as malondialdehyde (MDA) [73,74]. MDA causes a reduction of cell membrane fluidity and function [75]. ROS cause oxidative damage of proteins by direct interaction either on amino acid residues or cofactors or by indirect oxidation via lipid peroxidation end products [76,77]. Likewise, ROS target pyrimidine and purine bases, as well as the deoxyribose moiety of genomic and mitochondrial DNA, causing cellular damage such as strand breakage, nucleotide removal, and DNA-protein binding. Extensive damage that cannot be corrected by cellular DNA repair could result in permanent impairment followed by apoptosis [78]. 

As far as islet beta cells are concerned, they are highly susceptible to ROS-mediated damage because of insufficient amounts of antioxidative compounds such as glutathione, and the naturally low expression of antioxidative enzymes such as the mitochondrial SOD (Mn-SOD), cytoplasmic Cu/Zn SOD, glutathione peroxidase (GPx), and catalase [79]. Several examples also illustrate the critical role of antioxidative defenses in the vascular system in diabetes. For example, cardiomyocytes in diabetes overexpress SOD or catalase, protecting cardiac mitochondria from extensive oxidative damage. SOD also prevents morphological abnormalities in diabetic hearts, correcting the aberrant contractility [80,81]. Two emerging crucial regulators of antioxidative stress responses are the uncoupling protein 2 (UCP2) and the transcription factor NRF2 (NFE2L2). UCP2, originally thought to function in adaptive thermogenesis similar to UCP1, is now considered to be primarily a regulator of ROS generation in mitochondria. UCP2 is a proton channel protein localized on the inner mitochondrial membrane that reduces the electrochemical gradient on both sides of the membrane, decreases ROS production, and protects against oxidative damage in mitochondria [82]. UCP2 has a critical role in the regulation of glucose homeostasis and in oxidative stress-mediated vascular diseases [83,84]. As for NRF2, it controls the transcription of key components of many antioxidative responses by binding to antioxidant response (ARE) elements in the promoter regions of target genes such as members of the glutathione and thioredoxin antioxidant systems and NAPDH (nicotinamide adenine dinucleotide phosphate) regeneration [85]. NRF2-mediated antioxidative responses are dysfunctional in diabetes [86] and dysregulation of the NRF2 redox pathway affects healing of diabetic wounds [87]. 

## 4. Oxidative Stress Generated by T Cell-Mediated Recognition of Beta Cells

T1D is an autoimmune disease characterized by T cell-mediated recognition and destruction of insulin-producing beta cells [88]. The beta cells are destroyed during the inflammatory phase known as insulitis. Insulitis is a significant component of T1D pathology and is characterized by infiltration of islets by immune and inflammatory cells. The leucocytic infiltration in insulitis is relatively subtle and transient, and therefore is detected mostly in cases with recent onset of the disease (less than one year [89]. There is limited knowledge about autoreactive T cells and autoantigens involved in the development of T1D. A primary autoantigen that activates autoreactive T cells is insulin [90]. Current views on T1D onset suggest that autoimmune destruction by insulitis is secondary to primary invasion of macrophages and dendritic cells activated by intercellular ROS from resident pancreatic phagocytes. Stimulated macrophages and dendritic cells will induce inflammatory genes and carry beta cell antigens specifically to lymph nodes, where T cells are activated. The activated T cells will specifically destroy beta cells through proinflammatory cytokine insults and more intracellular ROS formation [91]. So far, there is no cure for autoimmune T1D. Treatment is mostly focused on intensive insulin therapy aiming at tight glycemic control, which can significantly reduce debilitating long-term complications. There is a genetic predisposition for T1D. The strongest associations point at HLA class II, specifically haplotypes DRB1and DQB1 [92]. Although the autoreactive antigens and self-reactive T cells involved in autoimmune attack in T1D are well documented, the mechanism is not yet completely understood, however, the contribution of ROS and proinflammatory cytokines in beta cell death is fully substantiated [93]. The immune-mediated recognition of beta cells by autoreactive T cells and cytotoxic CD8T cells generates ROS and proinflammatory cytokines, inducing beta cell destruction and enhancing the effector response of islet-specific self-reactive CD4 T cells and cytotoxic CD8 T cells [94]. The proinflammatory milieu includes cytokines such as INFg, TNFa, IL-6, IL-12p70 and IL-1b, and ROS [95]. The destructive effect of ROS is amplified by the generation of reactive nitrogen species (RNS), which are extremely toxic free radicals such as free radical nitric oxide (NO) produced by IL-1b in beta cells. The IL-1b activates the enzyme nitric oxide synthase (iNOS), catalyzing production of nitric oxide and ultimately the superoxide ROS [96],. NO interacts with superoxide to generate the highly destructive molecule peroxynitrite. Both NO derived RNS and ROS cause beta cell damage using different pathways [97]. It is important to emphasize that an unbalanced ratio of oxidative to antioxidative events is what causes free radical toxicity. This has been illustrated by a recent study showing the dual role, protective or toxic, of NO in beta cells [98]. As stated above, insulitis and beta cell destruction are the crucial components of T1D pathology, but these are observed only in a limited proportion of islets at any given time, even at the time of diagnosis. Other factors, such as intercellular oxidative stress, precede insulitis [99]. This raises the possibility that in addition to the immune-mediated damaging effect of insulitis, a high level of dysfunction of beta cell contributes to T1D pathology as well. Interestingly, the lipid peroxidation, and oxidative stress detected by the presence of malondialdehyde in plasma of nondiabetic first degree relatives of the patients with T1D [100] supports the observation that oxidative stress can be clinically detected before the onset of diabetes.

## 5. Oxidative Stress and Metabolic Syndrome and Insulin Resistance in T2 Diabetes

T2D is currently considered a metabolic and inflammatory disease closely associated with metabolic syndrome, a group of conditions such as high blood pressure, glucose intolerance, insulin resistance, obesity, and dyslipidemia [101]. In many cases, a pre-T2D condition known as pre-diabetes is the prelude to the development of the disease. Pre-diabetes is characterized by impaired glucose tolerance and a state of mild hyperglycemia, not high enough to be diagnosed as diabetes, but leading to glucose intolerance. In addition, the main features of pre-diabetes are metabolic abnormalities similar to T2D, with essential roles of proinflammatory cytokines and free fatty acids (FFA), which are elevated in obesity and T2D as well. These factors initiate oxidative stress-mediated pathways, eventually resulting in beta cell dysfunction, impaired insulin secretion, and insulin resistance of peripheral tissue. Many studies indicate that oxidative stress originates before hyperglycemia, which in turn significantly contributes to the later complications of T2D (similar to those of T1D), such as vascular damage, retinopathy, nephropathy, and neuropathy [102]. In vitro and in vivo studies have indicated that the major oxidative stress-mediated pathways activated by hyperglycemia and ROS are JNK/SAPK, p38 MAPK, NF-kB, and the hexosamine biosynthetic pathway [103]. The first two, JNK/SAPK and p38 MAPK, contribute to the development of insulin resistance via direct and indirect phosphorylation of serine and threonine residues of insulin receptors [104,105]. Numerous studies link transcription factor NF-kB with regulation of gene-associated complications of diabetes [106]. In addition, hyperglycemia and oxidative stress mediate their actions through other signaling pathways such as advanced glycation end products (AGEs). AGEs refer to a group of heterogeneous compounds formed by the Maillard reaction process that involves the non-enzymatic glycation of proteins, lipids, and nucleic acids by reducing sugars and aldehydes. AGEs function through the multiligand immunoglobulin superfamily receptor for advanced glycation end products (RAGEs). The AGE compounds directly affect proteins of the mitochondrial respiratory chain to generate reactive oxygen species (ROS) [107]. AGE and RAGE are involved in diabetes vascular pathologies as well [108]. They also activate production of the second messenger signaling lipid diacylglycerol leading to activation of several isoforms of the protein kinase C (PKC). Isoforms of PKC are implicated in generating insulin resistance [109,110,111]. Last, but not least, AGE increases utilization of the polyol pathway that will decrease the cofactor NAPDH, and therefore directly affects the production of antioxidative glutathione [112,113]. As described above, multiple signaling pathways contribute to oxidative stress-mediated damage leading to T2D. Therefore, dysregulation of miRNAs controlling these pathways can certainly contribute to development and persistence of diabetes. 

## 6. MicroRNAs in Diabetic Oxidative Stress 

We reviewed research articles in PubMed, primarily focusing on studies describing changes in the expression of miRNAs due to oxidative stress in the context of diabetes and their target components controlling mechanism of oxidative stress homeostasis. 

This review does not include studies dealing with miRNAs induced by proinflammatory cytokines generated by T1D autoimmune attack on beta cells. Thorough reviews have been written on this topic [46,114,115,116]. Table 1 lists the miRNAs reported as having an effect on oxidative stress in diabetes, the source of oxidative stress and the observed effect, target tissue or organ, and target genes. A few miRNAs, with known target tissue but unknown gene targets are included as well. Ten miRNAs identified in Table 1, overlap with a previous in silico analysis of miRNAs in human cells regulated in vitro by oxidative stress [117]. These are let-7f, miR-9, miR-16, miR-21, miR-22, miR-29b, miR-99a, miR-141, miR-144, and miR-200c. In order to make this overview of miRNAs and their targets in oxidative stress and diabetes easy to follow, we organized the miRNAs by their function in the affected tissues and organs.

### 6.1. Vascular Endothelial Cells, Diabetic Cardiomyopathy, and Muscle

MiR-21 is a miRNA related to diabetes. The expression of miR-21 is increased in the plasma of patients with impaired glucose tolerance and with T2D [150]. It has been proposed that circulating extracellular vesicles carrying miR-21 could be used as a marker of developing type 1 diabetes [175]. It has been found that miR-21 increases susceptibility to oxidative stress induced by fluctuating glucose levels in primary pooled human umbilical vein endothelial cells (HUVECs), by targeting genes regulating homeostasis of intracellular ROS, such as KRIT1, NRF2, and SOD2 [151]. A reduced expression of miR-21 protects against cardiac remodeling in diabetic cardiomyopathy (DCM). An in vivo experiment in mice confirmed, that suppression of miR-21 stimulates the nuclear hormone receptor PPAR (peroxisome proliferator activated receptor), known to regulate homeostasis in response to glucose and lipid levels. The PPAR initiates nuclear translocation of NRF2, and thus the antioxidative response of NRF2 protects from DCM [152]. MiR-21 also regulates the signaling pathway of the intracellular AGE–RAGE interaction and targets TIMP3, an inhibitor of extracellular matrix degradation in diabetic neuropathy [176]. 

Similarly, in a rat model of DCM, the expression of miR-503 is increased in myocardial cells and has a deleterious role by targeting NRF2 and antioxidant response element (ARE) signaling pathway as well [128]. The cluster of miR-200 is an important player in oxidative response in diabetes [177]. It is formed by the following five evolutionary conserved miRNAs: miR-200a, miR-200b, miR-200c, miR-141, and miR-429. These miRNAs can be grouped according to their seed sequences into subgroup I, miR-200a and miR-141 (AACACUG), and subgroup II composed of miR-200b, miR-200c, and miR-429 (AAUACUG), suggesting that miRNAs in each subgroup will target different genes. Several reports indicate that the miR-200 family has a role in the development of endothelial inflammation present in diabetic vascular complications and cardiovascular diseases. In many instances, the action of miR-200 is via targeting the (zinc finger E-box-binding homeobox) ZEB1. ZEB1 has a role in epithelial–mesenchymal transition (EMT) [141] and is associated with the inhibition of apoptosis. The thioredoxin-interacting protein, TXNIP, is induced in vivo by hyperglycemia and it inhibits the antioxidative function of thioredoxin resulting in accumulation of reactive oxygen species, cellular stress, and induction of the miR-200 family which induces apoptosis through inhibition of ZEB1. Likewise, inhibition of miR-200c restores endothelial function in diabetic mice through upregulation of ZEB1 [177], and in HUVEC under oxidative conditions miR-200 expression is increased which suppress ZEB1 causing apoptosis. Overexpression of ZEB1 in the cells reversed the effect [178]. Downregulation of ZEB1, by miR-200a/b/c and miR-429, contributes to activation of proinflammatory genes in vascular smooth muscle cells of diabetic mice [146]. Furthermore, the miR-200 family negatively regulates beta cell survival in type 2 diabetes in vivo. Overexpression of miR-200, in mice, causes beta cell death and is sufficient to render T2D lethal [179]. 

In addition, the family of miRNA-200 has been reported to exhibit a protective effect in diabetic oxidative stress by targeting high glucose-induced O-linked N-acetylglucosamine transferase (OGT), whose enzymatic activity is associated with diabetic complications, and endothelial inflammation in mice with diabetes. Experiments with human aortic endothelial cells (HAEC) confirmed miR-200 silencing OGT by direct binding to 3′UTR of mRNA [143].

Another important antioxidative gene that is regulated by the family of miR-200 is Sirtuin 1 (SIRT1) [177]. SIRT1 is NAD+-dependent deacetylase that controls histone chromatin proteins as well as non-histone proteins, many of them are transcription factors such as fork-head box O1 (FOXO)1. To date, seven sirtuins have been identified. They are associated with several cellular processes, such as energy balance, stress resistance, and insulin resistance. Some are located in the cytoplasm and others are located in the nucleus or mitochondria [180]. SIRT1, -2, -3, and -6 have a function in oxidative stress. By targeting SIRT1, endothelial nitric oxide synthase (eNOS) and FOXO1 miR-200 impairs their regulatory circuit and promotes ROS production and endothelial dysfunction [147]. It has been shown that miR-200 targets these three genes in vitro in HUVEC cells. The in vitro results were validated in three in vivo models of oxidative stress, human skin fibroblasts from old donors, femoral arteries from old mice, and a murine model of hindlimb ischemia [147].

In endothelial cells, SIRT1 is targeted by other miRNAs, increasing diabetes-related oxidative stress. Examples include the following: miR-34 induces endothelial inflammation by downregulating SIRT1 [156] and targeting SIRT1; miR-204 promotes vascular endoplasmic reticulum (ER) stress, inflammation, and dysfunction in mice; downregulation of miR-204 activates protection against ER stress through an increase of SIRT1 expression [157]; miR-106b targets SIRT1 in mouse insulinoma cell line NIT-1, rendering them vulnerable to hyperglycemia induced by 30mM glucose; and in vivo suppression of miR-106b increases expression of SIRT1 and reduces cardiovascular damage in diabetic mice [132]. 

Furthermore, it has been shown, in a mouse model of peripheral arterial disease, that the more abundant circulating form of unacylated ghrelin (UnAG) exerts its protective effect from ROS imbalance in endothelial cells via induction of miR-126, a known endothelial miRNA. By targeting vascular cell adhesion molecule 1 (VCAM1), miR-126 indirectly activates SIRT1 and SOD to induce resistance to oxidative stress [148]. 

MiR-9 plays a positive role in oxidative stress-mediated cardiomyopathy in T2D. In vitro experiments with immortalized cardiomyocyte culture and samples of failing heart tissue collected at the time of transplantation confirmed that downregulation of miR-9 in human cardiomyocytes results in higher expression of its target ELAV-like protein 1 (ELAVL1), a ubiquitously expressed RNA binding protein that stabilizes inflammatory mRNAs by binding to ARE domains and thus leading to cardiomyocyte death [167]. Another miRNA with a protective role in diabetic cardiomyopathy is miR-30c. MiR-30c targets PGC-1β, one of important coactivators of PPAR alpha and mitochondrial key regulator. Knockdown of PGC1 beta reduces excessive ROS and myocardial lipid accumulation which decreases cardiac dysfunction in diabetes [124].

Numerous studies report miR-29 family participation in oxidative stress-mediated inflammatory response in diabetes. The miR-29 family consists of three members divided into two clusters that are transcribed polycistronically; the miR-29a/b-1 cluster is localized on human chromosome 7 and the miR-29c/b-2 cluster on chromosome 1 [181]. The miR-29s are known to be regulated in multiple tissues. Hyperinsulinemia dramatically reduces their expression, while hyperglycemia induces it. Experiments with MIN6 insulinoma beta cell line determined that miR-29 targets a member of the BCL2 family, an antiapoptotic protein, the MCL1 (myeloid cell leukemia 1) (MCL-1) gene. Interestingly, in humans, repression of MCL1 is related to diabetes mellitus-associated cardiomyocyte disorganization [182]. Since circulating miR-29 has been reported in newly diagnosed T2D patients and, furthermore, upregulation of miR-29 expression contributes to development of the first stage of type 1 diabetes mellitus in the T1D model of NOD mice [170], there is the possibility that miR-29 regulates MCL1 at different stages of the disease. 

There are instances that indicate the miR-29 cluster family has a protective role against oxidative stress conditions. Its elevated expression has been associated with a compensatory mechanism for heart hypertrophy and fibrosis due to age increased oxidative stress, modulating targets such as DNA methylases and collagens [183]. A protective role in endothelial dysfunction in cardiometabolic disorders found in T2D has been reported. MiR-29 is upregulated in T2D arterioles to compensate for endothelial dysfunction. Specifically, miR-29 targets Lypla 1 (lysophospholipase I), a gene that negatively regulates production of NO, required for vasodilation. Lypla 1 depalmitoylates eNOS (nitric oxide synthase), reducing NO in endothelial cells [173].

The expression of miR-29a and miR-29c in skeletal muscle of patients with type 2 diabetes are upregulated which suppresses glucose and lipid metabolism possibly by targeting insulin receptor substrate 1 (IRS1) and phosphoinositide 3 kinase (PI3K). Both genes are involved in glucose insulin regulation, moreover they control lipid oxidation by targeting peroxisome activated receptor gamma coactivator1alpha (PGC1alpha). In vivo overexpression of miR-29 in mouse tibias anterior muscle resulted in a decrease of glucose uptake and glycogen content. MiR-29 acts as an important regulator of insulin stimulated glucose metabolism [171]. 

### 6.2. Retina Cells 

Oxidative stress and hypoxia cause retinopathy by induction of miR-7 that negatively regulates the RAPGEF3/EPAC-1 (rap guanine nucleotide exchange factor 3). EPAC-1 is an accessory protein for cAMP activation and stimulation for survival and growth in response to extracellular signals [134]. MiR-7-mediated decrease of EPAC1 expression results in endothelial hyperpermeability and loss of (endothelial nitric oxide synthase) eNOS activity in murine experimental retinopathy. EPAC-1 is associated with cAMP-induced vascular relaxation in endothelial cells via eNOS and amelioration of endothelial hyperpermeability induced by inflammatory mediators [134]. Development of retinopathy in T2D is associated with miR-15 as well. This miRNA is mostly found in the pancreas, where it plays an important role in beta cell insulin secretion. Interestingly, miR-15 has been detected in the plasma of T2D patients, where its amount corelated with the severity of the disease. Experiments with the rat beta cell line INS1 showed that the concentration of miR-15 in the cells increases when cultured in high glucose media. Coculture of INS1 insulinoma cells with Muller cells (retinal glial cells) showed a clear transfer of miR-15 into Muller cells, and the transfer was achieved by exosomes. The deleterious effect of miR-15 in the retina is via targeting AKT3, an isoform of the AKT gene (serine/threonine kinase 1). Loss of AKT3 in the tissue increases intracellular content of ROS, leading to cellular apoptosis. These results also prove that under pathological conditions some miRNAs can travel from tissue to tissue through exosome transfer [154]. Incidentally, persistent exposure to high glucose causes intracellular accumulation of insulin in beta cells mediated by suppression of the UPC2 gene by miR-15a. High glucose treatment for a short time induces miR-15a, while longer exposure suppresses the expression. It has been found that inhibition of UPC2 by miR-15a increases O_2_ consumption beta cell function and insulin synthesis [122]. 

Oxidative stress in retinal glial Muller cells induces upregulation of miR-365 causing damage by targeting TIMP3, the protein that inhibits matrix metalloproteinases and has antioxidative properties [130]. MiR-455-5p may have a positive role in diabetic retinopathy. Upregulation of miR-455-5p attenuates high glucose-triggered oxidative stress injury by targeting SOCS3 (suppressor of cytokine signaling 3) mRNA. SOCS3 downregulation decreases production of intracellular ROS, malondialdehyde (MDA) content, and NADPH oxidase 4 expression, while enhancing superoxide dismutase, catalase, and GPX activities [120]. 

### 6.3. Diabetic Wound 

Moreover, the miR-200 family has an effect on the pathology of diabetic skin ulcers by targeting the angiogenic factor angiopoietin 1 (ANGPT1), resulting in disrupted angiogenesis. In diabetic wound healing, hyperglycemia-mediated oxidative stress produces an unmodulated, persistent unfolded protein response (UPR), generating deficiency in inositol-requiring enzyme 1 (IRE1α), a primary UPR transducer that modulates expression of mRNAs and miRNAs. This deficiency leads to the upregulation of the miR-200 family and miR-466, both targeting ANGPT1. Angiogenesis may be rescued by upregulation of IRE-1a, which attenuates maturation of both miRNAs [166].

### 6.4. Kidney Tissues and Functions 

Another miRNA that interferes with ROS homeostasis in diabetes via targeting NRF2 is miR-27a. The adipokine omentin 1 restores renal function of type 2 diabetic db/db mice through suppression of miR-27a, which upregulates NRF2 and decreases oxidative stress [155]. NRF2/KEAP1 is a master antioxidant pathway regulating redox under nonstressed and stressed conditions. Under nonstressed conditions, NRF2 is anchored by a repressor KEAP1 in cytoplasm. A stressed situation releases KEAP1 and the stabilized NRF2 relocates to nucleus, where it binds to the antioxidant response element (ARE) activating transcription of antioxidant proteins [184]. In experiments with mice rendered diabetic with streptozotocin, hyperglycemia activates the polyol pathway in renal mesangial cells. The polyol pathway is involved in microvascular damage to retina in diabetes. On the one hand, activation of the polyol pathway increases the activity of aldose reductase which in turn decreases expression of miR-200a and miR-141. These miRNAs are regulators of KEAP-1. Their low expression enhances suppressive activity of KEAP-1 on NRF2. The suppressed transcription factor, NRF2, cannot activate transcription of antioxidant genes resulting in an increase of ROS and oxidative stress. On the other hand, aldose reductase deficiency in the renal cortex upregulates miR-200 and miR-141, which releases the KEAP-1 suppression of NRF2 and ameliorates the oxidative stress and downregulates TGF-beta, preventing kidney fibrosis [164]. The NRF2/KEAP1 pathway is also regulated in other organs under oxidative stress damage, such as in the pathological process of liver injury in T2DM. In this case, miR-233 targets KEAP1 allowing the released NFR2 to migrate to the nucleus and activate synthesis of antioxidative mRNAs and proteins such as SOD and HO-1 [125].

Endothelial dysfunction in cardiovascular disease is also affected by CKD (chronic kidney disease). CKD is caused by the accumulation of uremic toxin which upregulates miR-92a. The miRNA can be detected in the patient’s serum, which could be useful for diagnostic purposes. Uremic toxins generated oxidative stress results in downregulation of endothelial protective factors such as SIRT1 and eNOS [144]. At this time, it is not known if this is through direct or indirect regulation. Additionally, miR-92a is upregulated in diabetic aortic endothelium of C57BL-db/db mice and in renal arteries from human diabetic subjects. MiR-92a downregulates expression of heme oxygenase 1 (HO-1), an endothelial protective enzyme synthesized through NRF2 binding to the ARE sequence in the nucleus. The resulting oxidative stress impairs endothelium dependent relaxation. The suppression of miR-92 restores the endothelial function and the expression of HO-1 [145]. The expression of miR-25 in diabetic mouse kidneys and in human peripheral blood of patients with diabetes is much lower than in non-diabetic subjects. MiR-25 has a protective role in ROS-mediated diabetic kidney disease, by direct regulation of the Ras-related gene CDC42. The CDC42 gene belongs to the family of Rho small GTPases which are central regulators of actin reorganization and have a role in nephrotic pathogenesis. An increase of miR-25 expression represses glomerular fibrosis [139]. Some of the intracellular effects of ROS are mediated by regulation of the PTEN/PI3K/AKT pathway [185]. Blood samples and kidney tissue from diabetic subjects show downregulation of miR-25. Gain and loss of function performed with the human kidney cell line HK2 confirmed the crucial role of miR-25 protection against dysfunction and apoptosis of renal tubular epithelial cells. MiR-25 inhibits the apoptotic effect of hyperglycemia-mediated ROS in renal tubular epithelial cells by targeting PTEN. Knockout of PTEN activates the PI3K/AKT. PTEN is a dual protein and lipid phosphatase whose main substrate is phosphatidyl-inositol,3,4,5 triphosphate (PIP3). PTEN catalysis dephosphorization of PIP3 to PIP2 which represses the antiapoptotic signaling pathway of PI3k/AKT. Knockout of PTEN by miR-25 activates the AKT pathway ameliorating ROS and apoptosis [140]. Some miRNAs exert their antioxidative role by regulating the expression of UCP2 (uncoupling protein 2) which attenuates ROS activity in mitochondria. In HK2 (kidney cortex and proximal tubule cell line), it has been shown that miR-214 suppresses oxidative stress in diabetic nephropathy via the ROS/Akt/mTOR signaling pathway and enhancing UCP2 expression [121]. On the other hand, an experiment in a diabetic mouse model showed that miR-30e targets directly UCP2 in kidney cells, thus mediating the TGF-β1-induced epithelial-mesenchymal transition and kidney fibrosis [123]. In diabetic nephropathy, miRNA-29c contributes to the progression of the disease by regulating proinflammatory cytokines via targeting tristetraprolin (TTP) mRNA [172]. Experiments were performed in kidney tissues from DN patients and controls. TTP has anti-inflammatory effects by enhancing the decay of mRNAs bearing the adenosine/uridine-rich element (ARE) present in the 3′UTR of cytokine transcripts such as Il-6 and TNF alpha. Additional experiments with cultured podocytes confirmed the findings. Finally, miR-21, a diabetes-related miRNA, described above, has a role in diabetic nephropathy by regulating TIMP3, an inhibitor of extracellular matrix degradation [176], involved in mesangial expansion characteristic of diabetic nephropathy. 

### 6.5. Diabetic Neuropathy 

In the case of diabetic peripheral neuropathy, PKC activity is linked to a protective role of miR-25. MiR-25 downregulates production of AGE and RAGE, reduces activation of PKC, and reduces NAPDH oxidase activity probably via regulation of NOX4, an isoform of the NOX family. NOX4 protects vasculature against inflammatory stress. Experiments to clarify the protective role of miR-25 in diabetic neuropathy were done with sciatic nerve from db/db diabetic mouse model and BALB/c healthy counterparts. The conclusions were confirmed with cultured Schwann cells [138]. Modulation of the PTEN/AKT pathway is also critical to attenuate the oxidative stress mediated by extracellular amyloid-β (Aβ) peptides in diabetic neurotoxicity. Activation of the AKT pathway through direct targeting of PTEN by miR-302 attenuates amyloid beta induced toxicity in neurons and activated AKT signaling, which subsequently stabilizes NRF2 and synthesis of cytoprotective protein HO-1 [135]. 

Finally, as stated above, we have not included in this review the miRNAs involved in the oxidative stress caused by the effect of proinflammatory cytokines in beta cells. However, beta cells are also the target of other oxidative sources such as oxidized LDL (low density of lipoproteins). Oxidative stress induced the generation of oxidized LDL in hyperlipidemia conditions. Oxidized LDL enhances the activity of LPS (lysophosphatidylcholine) increasing the expression of miR-155-5p in murine pancreatic beta cells. MiR-155 targets MAFB (v-maf musculoaponeurotic fibrosarcoma oncogene family, protein B), enhancing the transcription of IL-6 that stimulates the production of GLP-1 in alpha cells, which suppresses glucagon secretion from alpha cells and stimulates insulin secretion from beta cells in a glucose-dependent manner. Through this mechanism, miR-155-5p improves the adaptation of beta cells to insulin resistance and protection of islets from stress [169].

### 6.6. Gestational Diabetes 

As discussed previously, the miR-29 family is regulated in multiple tissues. Although in most cases it has a deleterious and proinflammatory effect, in some organs the effect of miR-29 alleviates symptoms. In rats, miR-29b has a positive effect on gestational diabetes mellitus by targeting PI3K/Akt signal. Administration of miR-29 mimics reduced markers indicating oxidative stress, increased super oxide dismutase (SOD), catalase [165], and decreased malondialdehyde (MDA) in liver tissues of GDM rats [186]. Maternal diabetes and hyperglycemia dysregulate mitochondrial function through activation of protein kinase C (PKC) isoforms that have a role in the diabetic embryopathy. One of the isoforms of PKCα upregulates expression of miR-129-2, which targets the PGC-1α, the ligand of PPAR alpha (peroxisome proliferator activated receptor alpha). PGC1 alpha is a positive regulator of mitochondrial function and its downregulation by miR-129-2 mediates teratogenicity of hyperglycemia leading to NTDs (Neural tube defects in embryos) [131]. On the other hand, in the case of oxidative stress induced in embryo by maternal diabetes, inhibition of miR-27a increases NRF2 expression, which restores the homeostasis [129].

More recently, specific circular RNAs (circRNAs) interacting with miRNAs were identified in placentas from women with gestation diabetes mellitus that may regulate the AGE–RAGE interaction [174]. The circRNAs have their 5′ end and 3′ end covalently bond and are generated by a process known as back splicing, in which an upstream splice acceptor is joined to a downstream splice donor. They are expressed in various types of cells and tissues and, although little is known about their biological role, some act as gene regulators. In particular, several circRNAs have been described as acting as miRNA silencers or “sponges” by containing miRNA target sequences, in different type of cells including beta cells [187,188]. The differentially expressed circRNAs have been analyzed by Kyoto Encyclopedia of Genes and Genomes (KEGG) enrichment and circRNA–miRNA interaction, according to the sponge molecular interaction. The KEGG analysis predicted that circRNAs are likely to be involved in advanced glycation end products receptor for advanced glycation end products, AGE-RAGE, signaling pathways in diabetic complications. The expression of three circRNAs, circ-5824, circ-3636, and circ-0395, are downregulated in placentas of GDM. The circRNA–miRNA interaction analysis showed that miR-1273g-3p activated by acute glucose fluctuation is also involved in the progression of several complications caused by diabetes and it could be a potential gene of interest in GDM [174]. 

**Figure 2:**
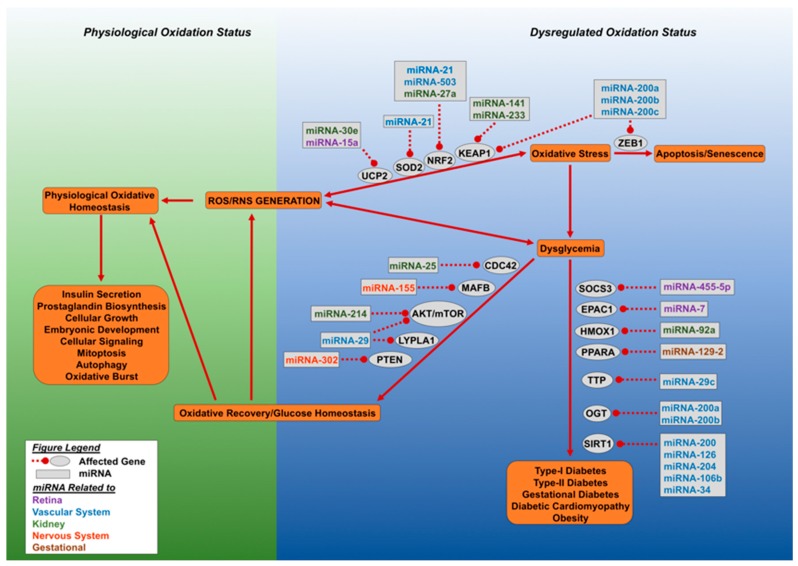
Dysregulated oxidative stress and microRNAs result in loss of glucose homeostasis. This figure outlines the effect of aberrant accumulation of cellular reactive oxygen species (ROS) and reactive nitrogen species (RNS). Cellular oxidative status is maintained by SOD2, NRF2, and UCP2, which allows for a spectrum of physiological functions carried out by the cell. Excessive ROS and RNS generation led to dysglycemia or cellular senescence. The miRNA molecules can target NRF2 (miRNA-21, miRNA-27a, miRNA-503, miRNA-233), SOD2 (miRNA-21), and UCP2 (miR-30e and miR-15a), leading to loss of oxidative regulation and the initiation of oxidative stress. Cellular oxidative stress can lead to either dysglycemia or cellular senescence. Cellular senescence is mediated by the inhibition of zinc finger E-box binding homeobox 1 (ZEB1) by miR-200 family miRNAs. Dysglycemia develops when O-linked β-N-acetylglucosamine transferase (OGT) and NAD-dependent deacetylase sirtuin-1 (SIRT1) are targeted by specific miRNAs. Oxidative stress driven dysglycemia rapidly initiates the expression of miRNA molecules which target suppressor of cytokine signaling 3 (SOCS3), exchange factor directly activated by cAMP 1 (EPAC1), and heme oxygenase (decycling) 1 (HMOX1), Peroxisome proliferator-activated receptor alpha (PPARA), mitochondrial uncoupling protein 2 (UCP2), and tristetraprolin (TTP), leading to decreased expression of these genes and the advance of diabetes. Alternatively, recovery can occur by miRNA directed targeting of genes involved in dysglycemia, they include: Cell division control protein 42 homolog (CDC42), V-maf musculoaponeurotic fibrosarcoma oncogene homolog B (MAFB), protein kinase B and mammalian target of rapamycin (AKT/mTOR), acyl-protein thioesterase 1 (LYPLA1) and phosphatase and tensin homolog (PTEN). Recovery of glucose homeostasis results in oxidative normalization and cellular homeostasis. Different colors of miRNA denote affected organ.

Figure 2 shows a scheme depicting the group of selected miRNAs described above and in Table 1 with their role in regulation of oxidative stress in diabetes

## 7. Conclusions

In diabetes, hyperglycemia induces intense oxidative stress that can no longer be modulated by the cellular antioxidative response, thus leading to accumulation of ROS. Overall, this process causes pancreatic beta cell dysfunction and unpaired glucose tolerance response, both of which have a deleterious effect on many types of cells and tissues. miRNAs have a critical role in the molecular mechanism involved in this process. Many of the studies reviewed here were performed in in vitro with animal cell lines or primary cells, in animal models (some in combination with tissues), some *in silico*, and a few cases in human tissues. It is expected that the development of new transgenic mice to study the role of miRNAs in oxidative stress will be useful to confirm or even discover novel potential targets and cellular pathways. However, the real challenge is the translation of all the in vitro, *in silico*, and animal model discovery to human diabetes. Although animal models, especially rodents, have been very useful for obtaining the basic information on the mechanism of several diseases, it is also true that the translation to human disease is not always straightforward. Specifically, many strategies were successful in treating autoimmune diabetes in rodent models, but none of them had been successful in treating human T1D. Furthermore, human basic and clinical research should aim at developing new strategies focusing on miRNAs and their target genes to cure diabetes and its complications. One of the emerging strategies is the use of a combination of human primary cells derived from human stem cell differentiation and organoid cultures plus genome editing alternatives to investigate the causes and role of miRNAs in oxidative stress in diabetes, as well as to screen for potential drugs to treat or alleviate its effects. However, it is important to remember that, currently, therapeutic approaches based on manipulation of miRNA expression are more effective in vitro than in vivo because of difficulties with specific delivery. As we have presented in this review, miRNAs are of variable nature, depending very much on the external and internal triggers. Therefore, it is of utmost importance to determine their specific targets and approach the treatment from that direction.

## Figures and Tables

**Table 1:** Selected PubMed articles describing miRNAs in diabetic oxidative stress.

Source of Oxidative Stress	Differentially Expressed miRNAs	Target Tissue/Organ	Target Gene	Reference
T2D	miR-203↓	Cardiac tissue	PIK3CA	[118]
T2D	miR-30e-5p↓	Kidney and vasculature	UCP2, MUC17, UBE2I	[119]
Diabetic retinopathy, hyperglycemia	miR-455-5p↓	Retinal epithelial cells	SOCS3	[120]
Diabetic nephropathy, hyperglycemia	miR-214↓	Kidney tissue	-	[121]
Insulin synthesis	miR-15a↑	Beta cells	UCP2	[122]
Kidney fibrosis	miR-30e↓	Tubular epithelial cells	UCP2	[123]
DCM	miR-30c↓	Cardiac tissue	PGC-1β	[124]
T2D	miR-233↓	Hepatic tissue	KEAP1	[125]
T1D, Diabetic nephropathy	miR-146a↓	Neural tissue, kidney tissue	-	[126,127]
DCM	miR-503↑	Cardiac tissue	NRF2	[128,129]
Diabetic Retinopathy	miR-365↓	Retinal tissue	TIMP3	[130]
Gestational Diabetes	miR-129-2↑	Murine neural tube	PGC-1α	[131]
Hyperglycemia	miR-106b↑	Pancreatic islets	SIRT1	[132]
Diabetic nephropathy	miR-106a↓	Murine neural tissue	ALOX15	[133]
Diabetic retinopathy	miR-7-5p↑	Retinal tissue	EPAC1	[134]
Diabetic neurotoxicity	miR-302↓	Neural tissue	PTEN	[135]
T2D	miR-17↓	Skeletal muscle	GLUT4	[136]
Diabetic retinopathy, hyperglycemia	miR-145↓	Retinal epithelial cells	TLR4	[137]
Diabetic nephropathy, hyperglycemia	miR-25↓	Neural tissue, kidney tissue	PTEN, CDC42	[138,139,140]
TXNIP overexpression	miR-200b↑	Beta cells	ZEB1	[141]
Diabetic mice	miR-200c↑	Vasculature	ZEB1	[142]
Diabetic Mice	miR-200a/b↓	Vasculature	OGT	[143]
DCM	miR-92a↑	Vasculature	HMOX1	[144,145]
T2D	miR-200b/c↑ and miR-429↑	Vasculature	ZEB1	[146]
T2D, T1D	miR-200c↑	Murine arteries	SIRT1, FOXO1, eNOS	[147]
Long-term diabetes	miR-126↑	Vasculature, skeletal muscles	SIRT1, SOD	[148]
T2D	miR-133a↓	Murine gastric smooth muscle cells	RhoA/Rho kinase	[149]
Hyperglycemia, T2D, T1D	miR-21↑	Vasculature, β-cells, Cardiac tissue	KIRT1, FOXO1, NRF2, SOD2, PPARA	[150,151,152]
T1D model	miR-200b↑	Murine retinal cells	OXR1	[153]
T2D	miR-15a↑	Plasma	AKT3	[154]
Diabetic embryopathy	miR-27a↑	Murine embryos, kidney tissue	NRF2	[129,155]
STZ-diabetic mice	miR-34a↑	β-cells, vasculature	SIRT1	[156]
Endothelial cells, vascular stress	miR-204↑	Vascular wall /endothelium in vivo	SIRT1	[157]
Cardiomyocytes apoptosis	miR-675↓	Vasculature	VDAC1	[158]
T1D, Diabetic retinopathy	miR-195↑	Cardiac tissue, β-cells	CASP3, MFN2	[159,160]
Gestational diabetes, hyperglycemia	miR-322↓	Murine Embryos, Neurons	TRAF3	[161]
T2D	miR-126↓	Vasculature	VEGFR2	[162]
T2D	miR-27b↓	Vasculature, wounds	SHC1, SEMA6A, TSP-1, TSP-2	[163]
Hyperglycemia, Polyol pathway	miR-200a-3p↑, miR-141-3p↑	Kidney tissue	KEAP1, TGFβ1/2	[164]
STZ mice	miR-1↓, miR-499↓, miR-133a/b↓ and miR-21↑	Cardiac tissue	ASPH	[165]
Persistent UPR IRE1α deficiency	miR-200↑, miR-466h-5p↑	Vasculature, wounds	ANGPT1	[166]
T2D, DCM	miR-9-5p↑	Retinal tissue	ELAVL1	[167]
T2D	miR-99a↑	Vasculature	IGF1R, MTOR	[168]
Hyperlipidemia	miR-155-5p↑	β-cells	MAFB	[169]
T1D NOD islets	miR-29c↑	β-cells	MCL1	[170]
T2D, glucose and lipid oxidation	miR-29↑	Skeletal muscle	-	[171]
Diabetic nephropathy	miR-29↑	Regulation of inflammatory cytokines	TTP	[172]
Diabetic heart T2D	miR-29↑	Cardio-metabolic disorders	Lypla 1	[173]
Gestational diabetes	Circular RNAs: circ-5824↓, circ-3636↓, circ-0395↓	Human placenta	(In silico analysis) AGE- and RAGE-related genes	[174]
